# *IRF5* variants and rheumatoid arthritis susceptibility in women from Central Mexico

**DOI:** 10.17305/bb.2025.12919

**Published:** 2025-09-08

**Authors:** Isaac Alberto López-Briceño, Guillermo Valencia-Pacheco, Isela Montúfar-Robles, Usman Zeb, Rosa Elda Barbosa-Cobos, Julian Ramírez-Bello

**Affiliations:** 1 Hematology Laboratory, Dr. Hideyo Noguchi Regional Research Center, Autonomous University of Yucatán, Mérida, Yucatán, Mexico; 2 Research Division, Juárez de México Hospital, Mexico City, Mexico; 3 Institute of Biotechnology and Genetic Engineering (IBGE), The University of Agriculture, Peshawar, KPK, Pakistan; 4 Rheumatology Service, Juárez de México Hospital, Mexico City, Mexico; 5 Clinical Research Directorate, Ignacio Chávez National Institute of Cardiology, Mexico City, Mexico

**Keywords:** *IRF5*, variants, risk, inflammatory, RA

## Abstract

Rheumatoid arthritis (RA) is a chronic autoimmune disease in which dysregulated interferon regulatory factor 5 (IRF5) may amplify pro-inflammatory pathways; prior genetic studies of *IRF5* single-nucleotide variants (SNVs) in RA are inconsistent across populations and have not included mestizo Mexicans or evaluated rs59110799 in RA. We aimed to test whether four *IRF5* SNVs (rs2004640G/T, rs2070197T/C, rs10954213G/A, rs59110799G/T) confer susceptibility to RA in women from Central Mexico. In a case-control study of 239 women with RA and 231 female controls (all self-identified Mexican-Mestizos, ≥3 generations), genotyping was performed by real-time PCR with TaqMan^®^ probes; 80% of samples were duplicated (100% concordance) and control genotypes conformed to Hardy–Weinberg equilibrium. Association was assessed under allelic and multiple genetic models using logistic regression adjusted for age and birthplace, with Bonferroni correction for 23 tests (α ═ 0.0022). Haplotype and linkage disequilibrium (LD) were analyzed with Haploview; putative functional effects were explored *in silico* (SNPinfo; GTEx). The minor alleles rs2004640T [OR=1.69, 95% CI 1.29–2.21; *P* ═ 1.2×10^−4^], rs2070197C [OR=1.85, 1.39–2.46; *P* ═ 2.0×10^−5^], and rs10954213A [OR=1.47, 1.12–1.93; *P* ═ 0.002] were associated with increased RA risk after correction. Genotype-based associations were observed for rs2004640 (codominant and recessive) and rs2070197 (codominant, dominant, recessive). rs59110799G/T showed no significant association after correction (dominant model OR=1.69, 1.15–2.48; *P* ═ 0.007). Nine haplotypes were identified; the haplotype carrying all four risk alleles (TCAT) was not associated, and two haplotypes with nominal signals (GCAG and TTGT) had control frequencies <1% and were excluded; variants were not in strong LD (*r*^2^<0.80). Our findings—providing the first evaluation of these *IRF5* variants in Mexican women and the first report of rs59110799 in RA—support a role for *IRF5* (rs2004640, rs2070197, rs10954213) in RA susceptibility in this Latin American population. Given the female-only design and moderate statistical power, replication and functional studies are warranted.

## Introduction

Rheumatoid arthritis (RA) is a chronic inflammatory autoimmune disease that affects approximately 0.5%–1% of the adult population globally. It is notably more prevalent in women than in men, with a ratio of 4:1 among younger individuals and 2:1 in older adults [1, 2]. RA is characterized by synovitis resulting from the activation of macrophages and fibroblast-like synoviocytes, along with the infiltration of lymphocytes, dendritic cells (DCs), neutrophils, mast cells, and monocytes into the synovial sublining. This pathological process is often accompanied by the production of autoantibodies, such as rheumatoid factor (RF) and anti-citrullinated peptide antibodies (ACPAs), which contribute to the ongoing inflammatory response. The inflammatory activity damages articular cartilage through the synthesis of pro-inflammatory cytokines, matrix metalloproteinases (MMPs), and other proteases. Additionally, the inflammatory environment promotes osteoclast activation, leading to bone erosion [3–8].

The etiology of RA remains unclear; however, its development is influenced by the interplay of epigenetic modifications, environmental factors, and hormonal influences in genetically predisposed individuals [9, 10]. Genome-wide association studies (GWAS) have identified over 100 risk loci associated with RA in European and Asian populations [11–15]. Among the genes linked to this autoimmune disorder (AD) is the interferon regulatory factor 5 (*IRF5*), which encodes a crucial transcription factor involved in regulating antiviral responses, type I interferon (IFN-I) production, and key biological processes such as apoptosis, cell cycle regulation, oncogenesis, and gene regulation in response to pathogen-derived signals. Moreover, IRF5 is instrumental in modulating the inflammatory response by mediating the production of pro-inflammatory cytokines (IL-6, IL-12, IL-23, and TNF), promoting macrophage polarization toward the M1 inflammatory phenotype, and regulating the responses of Th1 and Th17 lymphocytes [16–18]. In humans, IRF5 is expressed in isoforms that are specific to cell type, subcellular localization, and functional roles [19]. Additionally, certain single nucleotide variants (SNVs) within *IRF5* can lead to the expression of novel isoforms or increased levels of *IRF5* by enhancing the stability of its messenger RNA (mRNA) or protein, which contributes to susceptibility to systemic lupus erythematosus (SLE), Sjögren’s syndrome (SS), and multiple sclerosis. However, the role of IRF5 in susceptibility to RA remains unclear [20–23].

Previous studies in RA patients have investigated the *IRF5* rs2004640G/T, rs2070197T/C, and rs10954213G/A variants across various populations. The rs2004640G/T variant has been recognized as a significant risk factor for RA among Caucasian and Asian patients, particularly under the dominant genetic model (TT + GT vs GG) [24]. Additionally, this variant has shown an association with RA in patients from Korea and Tunisia, especially among those who are positive for ACPAs and the shared epitope (SE), as well as in individuals exhibiting joint erosion [25, 26]. In contrast, the rs2070197T/C and rs10954213G/A variants have been examined in Korean patients and the Han ethnic group in China, but they have not been established as risk factors for the disease [25, 27].

In mestizo Mexican populations, *IRF5* SNVs have not been studied in RA patients. In addition, no association has been reported for the rs59110799G/T variant in this AD. Previously, we identified this variant as a risk factor for SLE in women from central Mexico [28]. Furthermore, this SNV has been associated with primary SS (pSS) in Asian and European populations [29]. Since RA is a polygenic disease that shares susceptibility genes with other ADs, such as SLE and pSS [30–32], it is important to analyze *IRF5* variants in patients with this AD from Mexico. In addition, the role of *IRF5* variants on susceptibility to RA has been scarcely evaluated in ethnic groups other than Caucasians and Europeans. Thus, our study aimed to determine whether the *IRF5* rs2004640G/T, rs2070197T/C, rs10954213G/A, and rs59110799G/T SNVs are risk factors for RA in women from central Mexico.

## Materials and methods

### Study population

Our study involved 470 female participants from central Mexico, specifically from Mexico City, the State of Mexico, Morelos, Hidalgo, and Puebla. Participants were divided into two groups: 239 RA patients with an average age of 49.86 ± 12.36 years and 231 control subjects with no history of ADs or chronic inflammatory conditions, averaging 50.89 ± 4.44 years. Given that our study population comprised approximately 94 women diagnosed with RA for every 100 individuals—a proportion consistent with findings from other research groups in Mexico [33]—we chose to exclude male participants and concentrate exclusively on female patients.

Both patients and controls were over 18 years of age, self-identified as Mexican-Mestizos, and had at least three generations of ancestry. They were recruited at the Hospital Juárez de México (HJM) in Mexico City. All patients were classified according to the 2010 criteria established by the American College of Rheumatology (ACR) and the European League Against Rheumatism (EULAR) [34]. Individuals with concomitant ADs, infectious diseases (including human immunodeficiency virus, hepatitis C virus, severe acute respiratory syndrome coronavirus 2, influenza virus, or other viruses), genetic syndromes, and pregnant women were excluded from the study. Controls consisted of women without a family history of ADs or inflammatory diseases (including type 2 diabetes, obesity, hypertension, asthma, chronic urticaria, inflammatory bowel disease, allergies, or cancer), as well as those without infectious diseases or pregnancy history.

### Isolation of nuclear DNA from the buffy coat

Peripheral blood samples (6 mL, treated with EDTA) were collected to isolate nuclear DNA from the buffy coat using a modified version of the standard method that includes proteinase K digestion and salification [35]. The DNA from both patients and controls was quantified using the Thermo Scientific™ NanoDrop™ One/OneC spectrophotometer at 260/280 nm to assess concentration and purity. All DNA samples were subsequently diluted to a final concentration of 20 ng/µL for the allelic discrimination assay.

### Genotyping of *IRF5* SNVs

The genotypes of the *IRF5* SNVs rs2004640G/T (assay ID: C___9491614_10), rs2070197T/C (assay ID: C___2691236_10), rs10954213G/A (assay ID: C___31283335_10), and rs59110799G/T (assay ID: C___88886990_10) were determined through allelic discrimination using TaqMan^®^ probes (Applied Biosystems^®^, Foster City, CA) via real-time polymerase chain reaction (PCR). Amplification reactions were conducted using the StepOnePlus™ System thermocycler (Applied Biosystems^®^), adhering to the manufacturer’s protocol. The standardized PCR conditions included an initial incubation at 50 ^∘^C for 2 min, followed by denaturation at 95 ^∘^C for 10 min, and 40 cycles of denaturation at 95 ^∘^C for 15 s, with a combined annealing and extension step at 60 ^∘^C for 1 min.

To ensure precision, 80% of the samples underwent genotyping twice, achieving a reproducibility rate of 100%.

### In *silico* analysis

We used SNV function prediction (https://snpinfo.niehs.nih.gov/snpinfo/snpfunc.html) and the Genotype-Tissue Expression (GTEx) Portal (https://www.gtexportal.org/home/) to evaluate the possible *in silico* functional effect and their role as expression quantitative trait locus (eQTLs) of *IRF5* variants, respectively.

### Ethical statement

The protocol adhered to the ethical principles established in the Declaration of Helsinki and received approval from the Research, Ethics, and Biosafety Committee of HJM (HJM 017/22-I, July 2022–December 2025). All participants, both case and control, provided written informed consent prior to their enrollment in the study.

### Statistical analysis

The Hardy–Weinberg equilibrium (HWe) of the four *IRF5* SNVs was evaluated in our control group, with a significance threshold set at *P* < 0.05 to determine deviations from HWe. Allelic and genotypic frequencies, along with HWe, were calculated using the SNPstats© program (https://www.snpstats.net/start.htm). An association analysis between *IRF5* variants and RA susceptibility was conducted using STATA© software version 18, assessing various genetic models, including allelic, codominant, dominant, and recessive. To maintain statistical rigor, the Bonferroni correction was applied to adjust the *α* ═ 0.05 threshold based on the number of tests conducted (4 SNVs × 4 genetic models + 7 haplotypes = 16 + 7 = 23; 0.05/23 = 0.0022). Consequently, the significance threshold was established at *P* < 0.0022. Additionally, odds ratios (ORs) and *P* values were adjusted for covariates such as age and birthplace of cases and controls using logistic regression analysis in SPSS version 26. Haplotype construction and linkage disequilibrium (LD) analysis were performed using Haploview software version 4.2 (Broad Institute, 2020). The statistical power of the study was calculated with QUANTO© software version 1.2, utilizing parameters including the case-control ratio, a single-gene model, minor allele frequencies among controls for the four analyzed variants (rs2004640T = 38.53%, rs2070197C = 25.76%, rs10954213A = 48.27%, and rs59110799T = 23.38%), a recessive genetic model, an OR of 1.6, the prevalence of RA in Mexico City (1.0%) [36], and the sample sizes of cases and controls.

## Results

### HWe and statistical power

In our study, the genotype distribution of the four *IRF5* variants was found to be in HWe in the control group, with *P* values of 0.89 for rs2004640G/T, 0.86 for rs2070197T/C, 0.36 for rs10954213G/A, and 0.14 for rs59110799G/T. Furthermore, the statistical power for the analysis was determined to be 71.0% for rs2004640G/T, 64.5% for rs2070197T/C, 71.4% for rs10954213G/A, and 62.3% for rs59110799G/T.

### Association analysis of *IRF5* SNVs with RA

The allelic and genotypic frequencies of the *IRF5* rs2004640G/T, rs2070197T/C, and rs10954213G/A variants exhibited statistically significant differences between cases and controls, with minor allele frequencies of these variants being higher in patients than in the control group. Association analyses revealed that the alleles rs2004640T [OR = 1.69 (1.29–2.21), *P* ═ 0.00012], rs2070197C [OR = 1.85 (1.39–2.46), *P* ═ 0.00002], and rs10954213A [OR = 1.47 (1.12–1.93), *P* ═ 0.002] serve as risk factors for RA (Table 1). Furthermore, different genotypes of the *IRF5* variants rs2004640G/T and rs2070197T/C were associated with RA risk. Specifically, rs2004640G/T demonstrated an association under both codominant and recessive models, while rs2070197T/C was associated under codominant, dominant, and recessive models (Table 1).

**Table 1:** Genotypic and allelic frequencies of *IRF5* SNVs and association analysis in RA patients (*n* ═ 239) and controls (*n* ═ 231)

**Gene** **SNV**	**Model**	**Genotypes or alleles**	**RA** ***n* (%)**	**Controls** ***n* (%)**	**OR (95% CI)**	***P* value**
*IRF5* rs20046 40 G/T	Codominant	GG	66 (27.62)	88 (38.10)	–	–
		GT	107 (44.77)	108 (46.75)	1.45 (0.93–2.26)	0.093
		TT	66 (27.62)	35 (15.15)	2.95 (1.71–5.1)	**0.000 097**
	Allele	G	239 (50.00)	284 (61.47)	–	–
		T	239 (50.00)	178 (38.53)	1.69 (1.29–2.21)	**0.000 12**
	Dominant	GG	66 (27.62)	88 (38.10)	–	–
		TT+GT	173 (72.38)	143 (61.90)	1.81 (1.20–2.73)	0.005
	Recessive	GG+GT	173 (72.38)	196 (84.85)	–	–
		TT	66 (27.62)	35 (15.15)	2.35 (1.46–3.78)	**0.000 04**
*IRF5* rs20701 97 T/C	Codominant	TT	94 (39.33)	128 (55.41)	–	–
		TC	101 (42.26)	87 (37.66)	1.65 (1.1–2. 47)	0.016
		CC	44 (18.41)	16 (6.93)	3.85 (2.01–7.37)	**0.000 04**
	Allele	T	289 (60.46)	343 (74.24)	–	–
		C	189 (39.54)	119 (25.76)	1.85 (1.39–2.46)	**0.000 02**
	Dominant	TT	94 (39.33)	128 (55.41)	–	–
		CC+TC	145 (60.67)	103 (44.59)	1.99 (1.36–2.91)	**0.000 4**
	Recessive	TT+TC	195 (81.59)	215 (93.07)	–	–
		CC	44 (18.41)	16 (6.93)	3.05 (1.64–5.67)	**0.000 4**
*IRF5* rs10954 213 G/A	Codominant	GG	50 (20.92)	58 (25.11)	–	–
		GA	105 (43.93)	123 (53.25)	1.05 (0.65–1.71)	0.820
		AA	84 (35.15)	50 (21.65)	2.1 (1.22–3.59)	0.007
	Allele	G	205 (42.89)	239 (51.73)	–	–
		A	273 (57.11)	223 (48.27)	1.47 (1.12–1.93)	**0.002**
	Dominant	GG	50 (20.92)	58 (25.11)	–	–
		AA+GA	189 (79.08)	173 (74.89)	1.35 (0.86–2.13)	0.186
	Recessive	GG+GA	155 (64.85)	181 (78.35)	–	–
		AA	84 (35.15)	50 (21.65)	1.00 (0.98–1.00)	0.930
*IRF5* rs59110 799 G/T	Codominant	GG	116 (48.54)	140 (60.61)	–	–
		GT	100 (41.84)	74 (32.03)	1.68 (1.12–2.52)	0.011
		TT	23 (9.62)	17 (7.36)	1.74 (0.87–3.48)	0.113
	Allele	G	332 (69.46)	354 (76.62)	–	-
		T	146 (30.54)	108 (23.38)	1.45 (1.08–1.95)	0.012
	Dominant	GG	116 (48.54)	140 (60.61)	-	-
		TT+GT	123 (51.46)	91 (39.39)	1.69 (1.15–2.48)	0.007
	Recessive	GG+GT	216 (90.38)	214 (92.64)	–	–
		TT	23 (9.62)	17 (7.36)	1.41 (0.72–2.76)	0.311

Note: OR and *P* values were adjusted for the covariates age and place of birth of cases and controls. The associated alleles or genotypes of *IRF5* variants are highlighted in bold, with *P* values ≤ 0.0022. Abbreviations: IRF5: Interferon regulatory factor 5; SNV: Single nucleotide variant; RA: Rheumatoid arthritis; n: Number of individuals; OR: Odds ratio; CI: Confidence interval; *P* value: Probability value; rs: DbSNP reference SNP ID; GG: Homozygous G/G; GT: Heterozygous G/T; TT: Homozygous T/T; CC: Homozygous C/C; TC: Heterozygous T/C; GA: Heterozygous G/A; AA: Homozygous A/A.

Conversely, the allelic and genotypic frequencies of rs59110799G/T did not reveal statistically significant differences and were not identified as a risk factor for RA. However, a trend toward association was noted [OR = 1.69 (1.15–2.48), *P* ═ 0.007] under the dominant genetic model (GG vs. GT + TT). ORs, 95% confidence intervals (CIs), and *P* values for each *IRF5* variant in cases and controls are presented in Table 1.

### Haplotype and LD analysis

**Figure 1.**
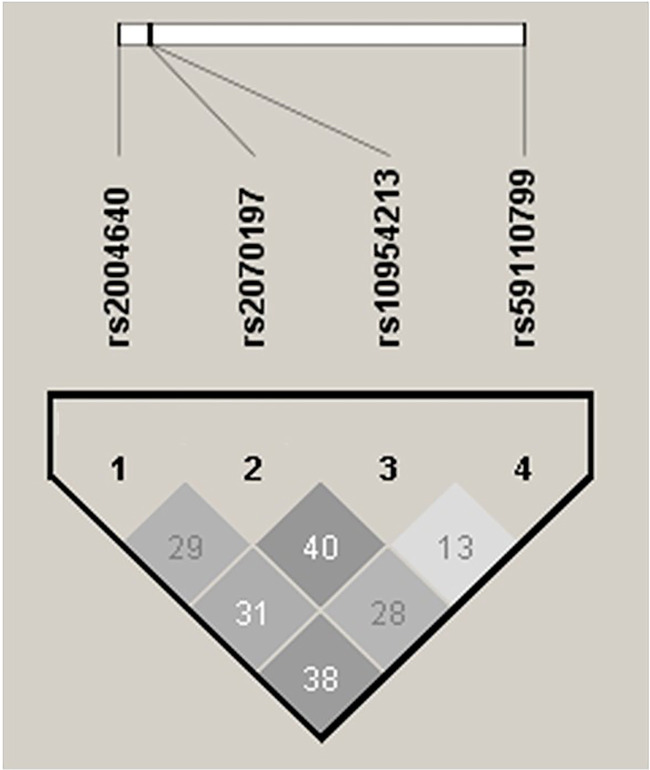
**Linkage disequilibrium plots in RA patients and controls.** All pairwise *r*^2^ values between *IRF5* SNVs were < 0.80, indicating weak linkage disequilibrium. Abbreviations: RA: Rheumatoid arthritis; IRF5: Interferon regulatory factor 5; SNV: Single nucleotide variant; *r*^2^: Squared correlation coefficient (LD measure); rs: Reference SNP identifier (dbSNP ID).

We identified nine haplotypes formed by combinations of alleles from the *IRF5* rs2004640G/T, rs2070197T/C, rs10954213G/A, and rs59110799G/T variants in both RA patients and controls. The TCAT haplotype, which carries the risk alleles of all four *IRF5* SNVs, showed no association with RA susceptibility (data not shown). After applying the Bonferroni correction, only two haplotypes, GCAG and TTGT, exhibited a strong association with RA susceptibility (data not shown). Each of these haplotypes contained two risk alleles: rs2070197T/C–rs10954213G/A and rs2004640G/T–rs59110799G/T, respectively. However, neither haplotype had a frequency exceeding 1% in the control group, leading to their exclusion from further analysis due to the low number of carriers (data not shown). Furthermore, we found that the four *IRF5* variants were not in LD (*r*^2^ < 0.80) (Figure 1).

## Discussion

In RA, the synovial membrane displays infiltration of activated immune cells, including macrophages, lymphocytes, and DCs, alongside resident cells such as chondrocytes, synovial fibroblasts, and osteoclasts. This cellular infiltration stimulates the production of pro-inflammatory cytokines, ultimately contributing to joint destruction. The synovial fluid of RA patients is characterized by elevated concentrations of cytokines (notably IL-1β, IL-6, and TNF), MMPs (including MMP-1, MMP-3, MMP-9, and MMP-13), and chemokines (such as IL-8, IP-10, MCP-1, and RANTES). Tumor necrosis factor (TNF), a pivotal cytokine in RA pathogenesis, is significantly elevated in these patients and is primarily secreted by Th1 lymphocytes and macrophages. TNF activates synovial fibroblasts, promotes epidermal hyperplasia, and facilitates the recruitment of inflammatory cells. Furthermore, TNF enhances the overexpression of cathepsins and MMPs, leading to the degradation of collagen and proteoglycans, which ultimately results in cartilage and bone destruction and joint erosion. Osteoclasts, activated by TNF, play a crucial role in disease progression by inducing synovial hyperplasia and angiogenesis [16, 37, 38].

A critical transcription factor involved in TNF synthesis is IRF5. In murine models, IRF5 has been shown to promote the polarization of synovial macrophages toward the M1 phenotype, leading to increased production of TNF and other pro-inflammatory cytokines, including IL-6, IL-12, and IL-23. This inflammatory milieu fosters the proliferation and differentiation of T-helper 1 (Th1) and T-helper 17 (Th17) lymphocytes, which, in turn, produce interferon-gamma (IFN-γ) and IL-17, respectively [18, 39, 40]. Notably, no correlation has been established between IFN-I and RA, although evidence suggests a cross-regulatory relationship between TNF and IFN-I [41]. Additionally, IRF5 is expressed in neutrophils; however, its precise role in RA pathogenesis remains inadequately understood. The importance of neutrophils is highlighted not only by their prevalence in the synovial fluid and pannus of active RA but also by their capacity to form neutrophil extracellular traps, which serve as a source of destructive enzymes and autoantigens [42].

The *IRF5* gene is situated on chromosome 7q32.1 and comprises a non-coding first exon that contains three alternative promoter regions (1A, 1B, and 1C), in addition to eight coding exons. Splicing at these alternative sites within exon one facilitates the production of various IRF5 isoforms. In humans, 11 isoforms have been identified, with expression levels differing based on cell type, subcellular localization, and functional role. Isoforms V1, V2, V4, and V5 are predominantly expressed in plasmacytoid DCs and macrophages, whereas V5 and V6 are found in peripheral blood mononuclear cells [16, 17, 19]. Research has demonstrated that SNVs in the *IRF5* gene can lead to the formation of novel IRF5 isoforms or enhance the expression of *IRF5* mRNA, potentially increasing the susceptibility to ADs such as RA [20–23]. In this study, we examined four specific *IRF5* SNVs (rs2004640G/T, rs2070197T/C, rs10954213G/A, and rs59110799G/T) in women diagnosed with RA from central Mexico to assess their contribution to the risk of developing this AD. It is noteworthy that certain autoimmune diseases, including SLE and pSS, exhibit a prevalence ratio of approximately nine affected women for every man [43, 44].

In Colombia, the ratio of women to men affected by RA is approximately 5.2:1 [45]. In Mexico, this ratio is even more pronounced; a study found that 91.7% of RA patients in the southern region were women, while another group reported 82.8% female patients [33]. In our cohort, 94% of RA patients were women, confirming findings from the southern Mexican population [33]. Due to the very small number of male participants in our study, we chose to exclude them from the analysis. This decision enabled us to achieve more robust and reliable results by concentrating exclusively on female patients. The predominance of women among RA patients may be influenced by genetic, environmental, hormonal, and other factors [46]. Our findings indicate that the *IRF5* rs2004640G/T, rs2070197T/C, and rs10954213G/A variants are significant risk factors for RA in Mexican women.

Our study indicates that the rs2004640G/T variant may serve as a susceptibility factor for the development of RA in women from central Mexico. This variant has previously been identified as a risk factor for RA in a meta-analysis that focused on Caucasian and Asian populations, particularly under the dominant genetic model [24]. Additional studies have shown that the rs2004640G/T variant is a risk factor in Korean patients, especially among those who are positive for ACPAs and SE alleles, as well as in Tunisian patients with ACPA positivity and joint erosion [25, 26]. However, it has not been recognized as a risk factor in Spanish, Japanese, and French populations [23, 47, 48].

Graham et al. [20] demonstrated that the rs2004640G/T variant, located two base pairs from the intron-exon boundary of exon 1B, creates a 5’ donor splice site in an alternative exon 1 of the *IRF5* gene, resulting in multiple *IRF5* transcripts. Individuals expressing exon 1B transcripts may produce IRF5 protein isoforms with varying levels of stability, affinity for transcriptional cofactors, and activation properties, leading to elevated *IRF5* mRNA levels. These isoforms could enhance the production of pro-inflammatory cytokines, such as IL-6, IL-12, and TNF, while also impairing the regulation of IFN-I, thereby contributing to chronic inflammation and joint tissue damage in RA patients.

Another variant associated with RA in our study population is rs2070197T/C. Previous research has primarily focused on Han Chinese patients, where no association with RA was identified [27]. Thus, our study represents the first report linking the *IRF5* rs2070197T/C variant to RA susceptibility. The precise functionality of this variant remains unclear; however, our earlier *in silico* analysis indicated that the rs2070197C allele creates a binding site for microRNA-24 (miR-24), which regulates the production of M1 and M2 macrophage cytokines. Overexpression of miR-24 has been shown to decrease pro-inflammatory cytokines while increasing M2 macrophage cytokines, which play a crucial role in immune regulation. Furthermore, this SNV functions as an eQTL, influencing *IRF5* expression in total blood [28]. These findings imply that the rs2070197T/C variant may modulate *IRF5* expression in the context of RA.

The rs10954213G/A variant, located in the 3’-UTR, exhibits significant functional activity. The risk allele, rs10954213A, introduces an alternative polyadenylation site, leading to increased mRNA expression and a shorter 3’-UTR. In contrast, the wild-type allele, rs10954213G, produces a 3’-UTR that is 1.5 kb long and contains two uridine-rich elements (AREs), which contribute to the RNA’s short half-life and rapid degradation. mRNAs with shorter 3’-UTRs typically demonstrate greater stability and higher transcript levels. This trend has been observed with the rs10954213A allele, which correlates with elevated IRF5 levels in cells stimulated with IFN-α. The presence of AREs in the 3’-UTRs of cytokine and growth factor genes underscores the importance of rapid mRNA turnover for genetic function [49]. In genetic studies of RA, this variant has not been recognized as a risk factor in Korean and Han Chinese populations [25, 27]. Nevertheless, our findings reveal an association with RA, underscoring the genetic variations present in the populations examined.

The rs59110799G/T variant has been previously identified as a risk factor for pSS in European and Asian populations through GWAS [29]. Furthermore, our earlier research found an association between this variant and SLE in a cohort of women from central Mexico; however, no such association was observed in women from Yucatan, indicating ancestral differences among Mexican populations [28]. In the present study, we noted a trend suggesting a potential association between this variant and RA; however, following Bonferroni correction, it was not recognized as a risk factor for this AD in Mexican women. *In silico* analysis indicates that rs59110799G/T does not directly affect the mRNA or protein levels of IRF5. Nevertheless, data from the GTEx portal suggest that it functions as an eQTL in total blood, implying its potential role in regulating *IRF5* gene expression [28].

RA, SLE, and pSS share common immune mechanisms, including chronic inflammation and susceptibility genes. Identifying genetic variants that modulate *IRF5* expression may yield valuable insights into genetic susceptibility markers for RA. Consequently, the identification of *IRF5* variants associated with this AD offers critical information regarding genetic markers that influence RA susceptibility within our population. Furthermore, this research underscores the shared RA susceptibility genes present across diverse ancestral populations.

In our study, we identified nine haplotypes formed by the combination of alleles from the rs2004640G/T, rs2070197T/C, rs10954213G/A, and rs59110799G/T variants. Seven of these haplotypes exhibited a frequency greater than 1% in the control group (data not shown). Notably, the haplotype consisting of the risk alleles from all four *IRF5* variants (TCAT) was not associated with RA. This study is the first to analyze haplotypes formed by these specific variants, as no previous research has addressed this particular combination. Existing studies have identified other haplotypes associated with RA that include some of the *IRF5* variants examined in our analysis. For instance, a study involving individuals of Han Chinese descent reported that the haplotype formed by exon 6 (deletion) and the rs2070197T, rs10954213A, and rs2004640T alleles is a significant risk factor for RA [27]. Additionally, research conducted on Spanish patients demonstrated that a haplotype composed of the rs729302A, rs2004640T, rs752637G, rs10954213A, rs13242262T, rs10488630A, rs10488631C, rs2280714A, and rs4731535T alleles also represents a risk factor for RA [23]. Furthermore, another report linked the haplotype formed by the rs3757385C, rs2004640T, and rs10954213A alleles to a cohort of RA patients with non-erosive disease [50].

The main limitations of our study include: a) the absence of clinical data and serological markers in RA patients, which hinders our ability to assess the association between *IRF5* variants and the clinical features of the disease. However, this does not preclude their potential involvement in these traits; b) the lack of AIMs limits our understanding of population stratification in our cases and controls. The inclusion of such markers would provide a clearer context for our findings. Consequently, the results regarding the association between the three *IRF5* variants and RA should be interpreted with caution; c) the low statistical power of our study due to the limited sample size. This constraint increases the risk of both false negatives and false positives, necessitating careful interpretation of our findings. Nonetheless, we believe our results contribute valuable evidence regarding Latin American populations and offer insights that warrant replication in larger, independent cohorts.

## Conclusion

Our study suggests that the *IRF5* rs2004640G/T, rs2070197T/C, and rs10954213G/A SNVs are risk factors for RA in women from Central Mexico. These findings provide additional evidence regarding the role of *IRF5* in susceptibility to RA, although further studies are needed to confirm these results and explore their functional mechanisms.

## Data Availability

The data that support the findings of this study are available from the corresponding author upon reasonable request.
